# Supplementary material for: Stability of the Inclusion Complexes of Dodecanoic Acid with α-Cyclodextrin, β-Cyclodextrin and 2-HP-β-Cyclodextrin
Source: Molecules. 2023 Mar 30;28(7):3113. doi: 10.3390/molecules28073113 (PMC10095696; doi:10.3390/molecules28073113)
Supplement: Supplementary file 1 [file molecules-28-03113-s001.zip › molecules-2310339-supplementary.pdf]

Stability of the inclusion complexes of dodecanoic acid with α-cyclodextrin, β-cyclodextrin and 2-HP-β-cyclodextrin

Zdzisław Kinart

Department of Physical Chemistry, Faculty of Chemistry, University of Lodz, Pomorska 163/165, 90-236 Lodz, Poland; zdzislaw.kinart@chemia.uni.lodz.pl

Table S1. Densities, viscosities and relative permittivities of pure water at temperatures T = (288.15 to 318.15)K.

T/K	ρ_0 /kg·m^{-3} [a]	$10^3 \cdot \eta$ /Pa·s [a]	ε_r [b]
283.15	999.700	1.130	83.95
288.15	999.100	1.138	82.07
293.15	998.205	1.002	80.21
298.15	997.047	0.890	78.40
303.15	995.651	0.797	76.62
308.15	994.038	0.719	74.89
313.15	992.224	0.653	73.19
318.15	990.223	0.596	71.53

[a] values for water recommended by the International Association for the Properties of Water and Steam (IAPWS) calculated on the online property calculator (https://web1.hszg.de/thermo_fpc/)

[b]values for water calculated according to the IAPWS recommendations (http://www.iapws.org/relguide/dielec.pdf)

Table S2. The value of molar concentration of salt C_{salt} [mol/dm^3], concentration of ligand C_{lig} [mol/dm^3], molar conductivity Λ [S·cm^2·mol^{-1}] for β-cyclodextrin with trans-cinnamic acid sodium salt in water at all tested temperatures at pressure p = 0.1 MPa.[a]

**sodium salt dodecanoic acid with α-cyclodextrin**										
T [K]			283.15	288.15	293.15	298.15	303.15	308.15	313.15	318.15
Nr	c_{salt} [mol/dm^3]	c_{lig} [mol/dm^3]	Λ_m [S·cm^2·mol^{-1}]	Λ_m [S·cm^2·mol^{-1}]	Λ_m [S·cm^2·mol^{-1}]	Λ_m [S·cm^2·mol^{-1}]	Λ_m [S·cm^2·mol^{-1}]	Λ_m [S·cm^2·mol^{-1}]	Λ_m [S·cm^2·mol^{-1}]	Λ_m [S·cm^2·mol^{-1}]
1.	0.00306	0.00000	46.3673	53.9311	61.8371	68.8551	75.8551	82.4197	87.1585	97.4252
2.	0.00302	0.00022	45.8558	53.6049	61.2358	67.7139	74.7139	81.0494	86.3254	95.9535
3.	0.00300	0.00032	45.6797	53.2061	60.9261	66.4466	73.4466	79.9996	85.7408	95.4152
4.	0.00297	0.00046	45.3341	52.8730	60.5521	65.2202	72.2202	79.0779	85.0545	94.5171
5.	0.00295	0.00059	45.0517	52.5449	60.1879	63.9763	70.9763	78.0714	84.2807	93.6089
6.	0.00291	0.00076	44.6472	52.1360	59.7004	62.7339	69.7339	76.5916	83.5244	92.7124
7.	0.00288	0.00090	44.3757	51.7832	59.3098	61.5588	68.5588	75.0721	82.7432	91.7931
8.	0.00285	0.00105	44.0327	51.4444	58.8821	60.4393	67.4393	73.6685	82.0511	90.7446
9.	0.00282	0.00120	43.7041	51.0464	58.3642	59.5847	66.5847	72.4074	81.3165	89.9063
10.	0.00278	0.00141	43.1879	50.4454	57.8724	59.0365	66.0365	71.4348	80.3239	88.5669
11.	0.00274	0.00161	42.8435	49.9867	57.2607	58.7173	65.7173	70.7572	79.2810	87.3150
12.	0.00270	0.00182	42.4606	49.5077	56.6228	58.5134	65.5134	70.3576	78.3524	86.0544
13.	0.00267	0.00199	42.0663	49.0600	56.1162	58.3841	65.3841	70.1632	77.3657	84.9440
14.	0.00263	0.00221	41.7411	48.6917	55.5630	58.2750	65.2750	69.9821	76.3964	84.0596
15.	0.00257	0.00249	41.4513	48.2900	54.9675	58.1947	65.1947	69.8296	75.4091	82.8867
16.	0.00251	0.00278	41.2379	48.0065	54.5344	58.1369	65.1369	69.7207	74.5524	82.0612
17.	0.00245	0.00309	41.1087	47.9446	54.2885	58.0984	65.0984	69.6516	74.0355	81.4904
18.	0.00239	0.00339	41.0466	47.8511	54.1508	58.0264	65.0264	69.5760	73.6723	81.0713
19.	0.00234	0.00366	41.1051	47.8743	54.0979	57.9517	64.9517	69.5208	73.5069	80.7227
20.	0.00228	0.00396	41.0893	47.8506	54.0093	57.9414	64.9414	69.4371	73.3432	80.4971
21.	0.00221	0.00429	41.0617	47.8739	54.0033	57.8920	64.8920	69.4140	73.1653	80.2429
22.	0.00215	0.00460	41.0855	47.8542	53.9557	57.8635	64.8635	69.3466	73.0055	79.9654
23.	0.00209	0.00489	41.0431	47.8006	53.9283	57.8237	64.8237	69.3352	72.8917	79.7934
24.	0.00203	0.00520	41.0808	47.7631	53.8857	57.7892	64.7892	69.2354	72.6195	79.5328
25.	0.00197	0.00548	41.0818	47.8292	53.8484	57.7623	64.7623	69.1586	72.5472	79.3186
26.	0.00192	0.00576	41.0877	47.8342	53.8012	57.7023	64.7023	69.0054	72.3917	79.2475
27.	0.00186	0.00606	41.0371	47.9207	53.7936	57.6533	64.6533	68.9723	72.3087	78.9683
28.	0.00180	0.00636	41.0113	47.8029	53.7815	57.6124	64.6124	68.8932	72.1597	78.7736

[a] Standard uncertainties are $u(T)$ = 0.01 K, $u(p)$ = 0.05 MPa, $u(c) = 10^{-4} \cdot c$, and the combined expanded uncertainty is $U_c(\Lambda) = 0.0005 \cdot \Lambda$ (level of confidence = 0.95).

Table S3. The value of molar concentration of salt C_{salt} [mol/dm³], concentration of ligand C_{lig} [mol/dm³],, molar conductivity Λ [S·cm²·mol⁻¹] for -β-cyclodextrin with dodecanoic acid sodium salt in water at all tested temperatures.

sodium salt dodecanoic acid with β -cyclodextrin										
T [K]			283.15	288.15	293.15	298.15	303.15	308.15	313.15	318.15
Nr	c_{salt} [mol/dm³]	c_{lig} [mol/dm³]	Λ_m [S·cm²·mol⁻¹]	Λ_m [S·cm²·mol⁻¹]	Λ_m [S·cm²·mol⁻¹]	Λ_m [S·cm²·mol⁻¹]	Λ_m [S·cm²·mol⁻¹]	Λ_m [S·cm²·mol⁻¹]	Λ_m [S·cm²·mol⁻¹]	Λ_m [S·cm²·mol⁻¹]
1.	0.00298	0.00000	45.2173	52.0673	58.2793	65.5795	73.8305	79.0645	85.7295	94.8508
2.	0.00294	0.00023	44.7058	51.5558	57.7678	65.0680	73.3190	78.5530	85.2180	94.3393
3.	0.00292	0.00030	44.5297	51.3797	57.5917	64.8919	73.1429	78.3769	85.0419	94.1632
4.	0.00289	0.00044	44.1841	51.0341	57.2461	64.5463	72.7973	78.0313	84.6963	93.8176
5.	0.00287	0.00059	43.9017	50.7517	56.9637	64.2639	72.5149	77.7489	84.4139	93.5352
6.	0.00284	0.00073	43.4972	50.3472	56.5592	63.8594	72.1104	77.3444	84.0094	93.1307
7.	0.00281	0.00088	43.2257	50.0757	56.2877	63.5879	71.8389	77.0729	83.7379	92.8592
8.	0.00278	0.00105	42.8827	49.7327	55.9447	63.2449	71.4959	76.7299	83.3949	92.5162
9.	0.00276	0.00118	42.5541	49.4041	55.6161	62.9163	71.1673	76.4013	83.0663	92.1876
10.	0.00272	0.00139	42.0379	48.8879	55.0999	62.4001	70.6511	75.8851	82.5501	91.6714
11.	0.00268	0.00160	41.6935	48.5435	54.7555	62.0557	70.3067	75.5407	82.2057	91.3270
12.	0.00264	0.00180	41.3106	48.1606	54.3726	61.6728	69.9238	75.1578	81.8228	90.9441
13.	0.00260	0.00201	40.9163	47.7663	53.9783	61.2785	69.5295	74.7635	81.4285	90.5498
14.	0.00259	0.00218	40.5911	47.4411	53.6531	60.9533	69.2043	74.4383	81.1033	90.2246
15.	0.00251	0.00248	40.3013	47.1513	53.3633	60.6635	68.9145	74.1485	80.8135	89.9348
16.	0.00246	0.00277	40.0879	46.9379	53.1499	60.4501	68.7011	73.9351	80.6001	89.7214
17.	0.00240	0.00306	39.9587	46.8087	53.0207	60.3209	68.5719	73.8059	80.4709	89.5922
18.	0.00235	0.00336	39.8966	46.7466	52.9586	60.2588	68.5098	73.7438	80.4088	89.5301
19.	0.00229	0.00369	39.9550	46.8050	53.0170	60.3172	68.5682	73.8022	80.4672	89.5885
20.	0.00224	0.00395	39.9393	46.7893	53.0013	60.3015	68.5525	73.7865	80.4515	89.5728
21.	0.00218	0.00425	39.9117	46.7617	52.9737	60.2739	68.5249	73.7589	80.4239	89.5452
22.	0.00213	0.00454	39.9355	46.7855	52.9975	60.2977	68.5487	73.7827	80.4477	89.5690
23.	0.00208	0.00481	39.8931	46.7431	52.9551	60.2553	68.5063	73.7403	80.4053	89.5266
24.	0.00201	0.00515	39.9308	46.7808	52.9928	60.2930	68.5440	73.7780	80.4430	89.5643
25.	0.00196	0.00543	39.9318	46.7818	52.9938	60.2940	68.5450	73.7790	80.4440	89.5653
26.	0.00190	0.00573	39.9377	46.7877	52.9997	60.2999	68.5509	73.7849	80.4499	89.5712
27.	0.00185	0.00601	39.8871	46.7371	52.9491	60.2493	68.5003	73.7343	80.3993	89.5406
28.	0.00181	0.00622	39.8613	46.7113	52.9233	60.2235	68.4945	73.7185	80.3735	89.5148

Table S4. The value of molar concentration of salt C_{salt} [mol/dm^3], concentration of ligand C_{lig} [mol/dm^3],, molar conductivity Λ [S·cm^2·mol^{-1}] for 2-HP-β-cyclodextrin with dodecanoic acid sodium salt in water at all tested temperatures.

sodium salt dodecanoic acid with 2-HP- β -cyclodextrin										
T [K]			283.15	288.15	293.15	298.15	303.15	308.15	313.15	318.15
Nr	c_{salt} [mol/dm^3]	c_{lig} [mol/dm^3]	Λ_m [S·cm^2·mol^{-1}]	Λ_m [S·cm^2·mol^{-1}]	Λ_m [S·cm^2·mol^{-1}]	Λ_m [S·cm^2·mol^{-1}]	Λ_m [S·cm^2·mol^{-1}]	Λ_m [S·cm^2·mol^{-1}]	Λ_m [S·cm^2·mol^{-1}]	Λ_m [S·cm^2·mol^{-1}]
1.	0.00302	0.00000	44.3762	51.0412	56.9229	64.3586	71.0984	77.8936	84.4169	92.0083
2.	0.00299	0.00020	43.8596	50.3616	56.1405	63.0593	69.9471	76.9880	83.0188	90.9240
3.	0.00297	0.00033	43.5922	50.0081	55.6119	61.7282	69.3135	76.3365	82.3231	90.1554
4.	0.00295	0.00052	43.2127	49.5715	55.0949	60.4869	68.4117	75.4494	81.6273	89.3318
5.	0.00294	0.00061	42.9049	49.2003	54.5788	59.1901	67.8271	75.0263	80.6531	88.4962
6.	0.00292	0.00074	42.5393	48.8021	54.0514	57.9340	66.9642	74.4679	79.8199	87.6191
7.	0.00290	0.00088	42.2326	48.2434	53.4236	56.8068	66.4194	73.8350	78.8874	86.8030
8.	0.00288	0.00102	41.7722	47.8890	52.8848	55.7046	65.5933	73.1282	78.3027	85.8701
9.	0.00287	0.00113	41.4772	47.5172	52.3592	54.8014	64.8884	72.5909	77.3413	84.9845
10.	0.00283	0.00141	40.9418	46.9407	51.5872	54.0595	63.8745	71.1932	76.2034	82.5445
11.	0.00281	0.00163	40.4607	46.3527	50.8602	53.6007	62.9219	70.1248	75.1625	81.3427
12.	0.00278	0.00184	40.0224	45.8560	50.1457	53.3147	62.0531	69.1486	73.9357	80.3113
13.	0.00275	0.00204	39.5545	45.2348	49.4452	53.0856	61.2729	68.2963	72.7772	79.2875
14.	0.00272	0.00231	39.1549	44.8061	48.7889	52.9267	60.6235	67.1964	71.9173	78.0327
15.	0.00267	0.00267	38.4597	44.1491	48.0061	52.7792	59.8774	66.0144	70.5765	76.8927
16.	0.00264	0.00293	38.1375	43.6424	47.3170	52.6703	59.3366	65.3646	69.4289	76.1261
17.	0.00259	0.00329	37.8252	43.2597	46.8021	52.5872	58.9208	64.7589	68.6211	75.6232
18.	0.00256	0.00355	37.6438	43.0187	46.4067	52.4999	58.6360	64.4852	68.0692	75.1438
19.	0.00252	0.00387	37.5277	42.7813	46.0384	52.4337	58.4220	64.2090	67.5090	74.7990
20.	0.00248	0.00417	37.3514	42.6102	45.7419	52.3615	58.2329	64.0110	67.2861	74.4679
21.	0.00244	0.00448	37.1972	42.5235	45.5989	52.3083	58.0806	63.8468	67.0386	74.3791
22.	0.00240	0.00476	37.1246	42.4230	45.3808	52.2533	57.9387	63.7291	66.8139	74.1293
23.	0.00236	0.00506	37.0070	42.3289	45.1944	52.2352	57.8097	63.6188	66.6323	73.9686
24.	0.00233	0.00533	36.9897	42.2571	44.9888	52.2032	57.6940	63.5100	66.3799	73.7655
25.	0.00229	0.00564	36.9503	42.1919	44.8237	52.1895	57.5534	63.4215	66.1903	73.7441
26.	0.00224	0.00602	36.9024	42.0642	44.5935	52.1721	57.5322	63.3306	65.9034	73.4474
27.	0.00182	0.00623	36.8565	42.0252	44.5023	52.1435	57.4393	63.3652	65.8736	73.4165

Table S5. Values of parameters A, B, and C for α-cyclodextrin, β-cyclodextrin and 2-HP-β-cyclodextrin with sodium salt of dodecanoic acid in water at all tested temperatures

	**A**	**B**	**C**
2-HP-β-cyclodextrin	216835	-1533.70	2.4638
β-cyclodextrin	102202	-828.20	1.3760
α-cyclodextrin	92268	-772.77	1.2956
